# Supplementary material for: Self-Medication for the Treatment of Abdominal Cramps and Pain—A Real-Life Comparison of Three Frequently Used Preparations
Source: J Clin Med. 2022 Oct 28;11(21):6361. doi: 10.3390/jcm11216361 (PMC9657472; doi:10.3390/jcm11216361)
Supplement: Supplementary file 1 [file jcm-11-06361-s001.zip › jcm-1952014-supplementary.pdf]

Online supplement to

A real-life comparison of three self-medication preparations for overlapping indications related to abdominal cramps and pain

Martin Storr, Harald Weigmann, Sabine Landes, Martin C. Michel

Supplemental Table S1: Perceived triggers of GI cramps and pain. Numbers are % of participants choosing a given option with multiple nominations being possible.

	**HBB**	**PLUS**	**PO**
n	496	381	282
Stress	53.4	46.2	58.5
Too little physical activity	21.9	22.7	23.4
Nutrition (e.g., too fatty, too sweet)	36.2	35.6	36.9
Food intolerance (e.g., lactose, gluten)	17.8	20.8	24.5
Heartburn	11.5	11.6	14.2
Bloating	31.4	41.2	48.2
Flatulence	17.2	23.5	30.9
Diarrhea	17.2	17.7	23.8
Sluggish bowels	12.3	19.8	23.8
Constipation	13.4	17.9	19.1
Unfavorable intestinal bacteria	6.5	7.4	15.6
Infection	5.3	6.1	5.7
Environmental pollution	2.6	1.8	2.8
Other	23.5	19.8	7.1
Don’t know	10.9	7.7	14.5

Supplemental Table S2: Effects of preparation, demographic, and baseline variables on improvement of impairment of work/daily chores on a 0-10 Likert scale, which was by 3.7, 2.9 and 3.5 points for HBB, PLUS and PO, respectively, in the overall group (see main Figure 2). Data are shown as effect size estimate with its 95% CI based on 1127, 383, 501, and 341 patients, respectively. These are absolute effect sizes for the categorical independent variables (preparations and gender) relative to the indicated reference group; they are values per year of age and per score of baseline values on the Likert scale for the continuous independent variables. N.d.: not determined because not an approved indication for the preparation.

	**Indication**			
	**GI cramps and pain**	**IBS**	**Bloating**	**Flatulence**
Preparation				
HBB	-0.1037 [-0.4171; 0.2096]	reference group	reference group	reference group
PLUS	reference group	n.d.	n.d.	n.d.
PO	-0.3845 [-0.7448; -0.0243]	-0.3420 [-0.8719; 0.1878]	-0.2063 [-0.6072; 0.1945]	-0.1278 [-0.6392; 0.3837]
Gender				
Female	reference group	reference group	reference group	reference group
Male	0.0214 [-0. 0.297; 0.3396]	0.1797 [-0.3883; 0.7478]	0.3062 [-0.1602; 0.7726]	0.3483 [-0.2187; 0.9152]
Age	-0.0183 [-0.0260; -0.0106]	-0.0122 [-0.0273; 0.0030]	-0.0195 [-0.0318; -0.0071]	-0.0141 [-0.0287; 0.0004]
Baseline symptom severity	0.0464 [-0.0300; 0.1227]	0.1047 [-0.0473; 0.2568]	0.0938 [-0.0235; 0.2111]	0.0646 [-0.0742; 0.2035]
Baseline leisure impairment	0.4913 [0.4280; 0.5546]	0.5458 [0.4149; 0.6766]	0.5099 [0.4095; 0.6104]	0.5260 [0.4040; 0.6479]
Baseline sleep impairment	0.0069 [-0.0414; 0.0552]	-0.0357 [-0.1229; 0.0514]	-0.0112 [-0.0869; 0.0645]	-0.0221 [-0.1157; 0.0715]

<u>Supplemental Table S3:</u> Effects of preparation, demographic, and baseline variables on improvement of impairment of leisure activities on a 0-10 Likert scale, which was by 3.7, 3.8 and 3.4 points for HBB, PLUS and PO, respectively, in the overall group (see main Figure 2). Data are shown as effect size estimate with its 95% CI in 1126, 384, 502, and 342 patients, respectively. These are absolute effect sizes for the categorical independent variables (preparations and gender) relative to the indicated reference group; they are values per year of age and per score of baseline values on the Likert scale for the continuous independent variables. N.d.: not determined because not an approved indication for the preparation.

	**Indication**			
	**GI cramps and pain**	**IBS**	**Bloating**	**Flatulence**
Preparation				
HBB	0.1073 [-0.1934; 0.4080]	reference group	reference group	reference group
PLUS	reference group	n.d.	n.d.	n.d.
PO	-0.3356 [-0.6813; 0.0100]	-0.3333 [-0.8448; 0.1782]	-0.1326 [-0.5314; 0.2662]	-0.3000 [-0.8055; 0.2055]
Gender				
Female	reference group	reference group	reference group	reference group
Male	0.3047 [-0.0009; 0.6104]	0.2416 [-0.3071; 0.7903]	0.3394 [-0.1248; 0.8036]	0.6154 [0.0551; 1.1757]
Age	-0.0069 [-0.0143; 0.0006]	-0.0085 [-0.0231; 0.0061]	-0.0061 [-0.0184; 0.0062]	0.0004 [-0.0140; 0.0148]
Baseline symptom severity	0.0978 [0.0260; 0.1696]	0.1282 [-0.0157; 0.2722]	0.1239 [0.0091; 0.2388]	0.0495 [-0.0872; 0.1862]
Baseline work/daily chore impairment	0.4484 [0.3912; 0.5056]	0.4132 [0.2996; 0.5267]	0.4199 [0.3272; 0.5127]	0.4012 [0.2907; 0.5116]
Baseline sleep impairment	0.0114 [-0.0349; 0.0577]	-0.0417 [-0.1261; 0.0427]	0.0123 [-0.0623; 0.0868]	0.0557 [-0.0349; 0.1462]

Supplemental Table S4: Effects of preparation, demographic, and baseline variables on improvement of impairment of sleep on a 0-10 Likert scale, which was by 2.8, 3.1 and 2.5 points for HBB, PLUS and PO, respectively, in the overall group (see main Figure 2). Data are shown as effect size estimate with its 95% CI based on 1130, 383, 502, and 341 patients, respectively. These are absolute effect sizes for the categorical independent variables (preparations and gender) relative to the indicated reference group; they are values per year of age and per score of baseline values on the Likert scale for the continuous independent variables. N.d.: not determined because not an approved indication for the preparation.

	**Indication**			
	**GI cramps and pain**	**IBS**	**Bloating**	**Flatulence**
Preparation				
HBB	-0.1252 [-0.4912; 0.2409]	reference group	reference group	reference group
PLUS	reference group	n.d.	n.d.	n.d.
PO	-0.3527 [-0.7733; 0.0680]	-0.1787 [-0.7365; 0.3792]	-0.1114 [-0.5671; 0.3443]	-0.0030 [-0.5944; 0.6003]
Gender				
Female	reference group	reference group	reference group	reference group
Male	0.0435 [-0.3292; 0.4162]	0.2523 [-0.3441; 0.8486]	0.4036 [-0.1251; 0.9323]	0.4243 [-0.2340; 1.0826]
Age	0.0007 [-0.0083; 0.0097]	-0.0007 [-0.0165; 0.0151]	-0.0018 [-0.0159; 0.0122]	0.0022 [-0.0147; 0.0192]
Baseline symptom severity	0.2181 [0.1307; 0.3055]	0.2428 [0.0874; 0.3983]	0.2733 [0.1427; 0.4039]	0.2740 [0.1145; 0.4336]
Baseline work/daily chore impairment	0.1586 [0.0549; 0.2623]	0.1734 [-0.0053; 0.3520]	0.0936 [-0.0684; 0.2557]	-0.0103 [-0.2086; 0.1881]
Baseline leisure impairment	0.1310 [0.0207; 0.2413]	0.1186 [-0.0801; 0.3173]	0.1869 [0.0150; 0.3589]	0.2859 [0.0749; 0.4969]
